# Evidence for oscillating circadian clock genes in the copepod *Calanus finmarchicus* during the summer solstice in the high Arctic

**DOI:** 10.1098/rsbl.2020.0257

**Published:** 2020-07-15

**Authors:** Lukas Hüppe, Laura Payton, Kim Last, David Wilcockson, Elizaveta Ershova, Bettina Meyer

**Affiliations:** 1 Institute for Chemistry and Biology of the Marine Environment, Carl von Ossietzky University of Oldenburg, 26111 Oldenburg, Germany; 2 Helmholtz Institute for Functional Marine Biodiversity (HIFMB) at the University of Oldenburg, 26111 Oldenburg, Germany; 3 Alfred Wegener Institute Helmholtz Centre for Polar and Marine Research, Department of Biosciences, Section Polar Biological Oceanography, 27570 Bremerhaven, Germany; 4 Scottish Association for Marine Science, Oban, Argyll PA37 1QA, UK; 5 Institute of Biological, Environmental, and Rural Sciences, Aberystwyth University, Aberystwyth SY23 3DA, UK; 6 Department for Arctic and Marine Biology, Faculty for Biosciences, Fisheries and Economics, UiT The Arctic University of Norway, 9019 Tromsø, Norway; 7 Shirshov Institute of Oceanology, Russian Academy of Sciences, Russian Federation, 36 Nakhimova Avenue, Moscow 117997, Russia

**Keywords:** Arctic, Midnight Sun, circadian clock, copepod, zooplankton, sea ice

## Abstract

The circadian clock provides a mechanism for anticipating environmental cycles and is synchronized by temporal cues such as daily light/dark cycle or photoperiod. However, the Arctic environment is characterized by several months of Midnight Sun when the sun is continuously above the horizon and where sea ice further attenuates photoperiod. To test if the oscillations of circadian clock genes remain in synchrony with subtle environmental changes, we sampled the copepod *Calanus finmarchicus,* a key zooplankter in the north Atlantic, to determine *in situ* daily circadian clock gene expression near the summer solstice at a southern (74.5° N) sea ice-free and a northern (82.5° N) sea ice-covered station. Results revealed significant oscillation of genes at both stations, indicating the persistence of the clock at this time. While copepods from the southern station showed oscillations in the daily range, those from the northern station exhibited an increase in ultradian oscillations. We suggest that in *C. finmarchicus*, even small daily changes of solar altitude seem to be sufficient to entrain the circadian clock and propose that at very high latitudes, in under-ice ecosystems, tidal cues may be used as an additional entrainment cue.

## 1. Introduction

Biological clocks are ubiquitous, ancient and adaptive mechanisms enabling organisms to track and anticipate environmental cycles and regulate biological processes accordingly. Recent work on *Calanus finmarchicus*, a key pelagic species in the northern Atlantic food web [1], revealed that *C. finmarchicus* possesses a functional circadian clock that might be involved in the timing of both diel vertical migration (DVM) [2] and seasonal events such as diapause [3].

The Arctic is characterized by strong seasonal fluctuations in photoperiod leading to permanent illumination during Midnight Sun and permanent darkness during Polar Night. Since circadian clocks of most organisms use the daily light/dark cycles as a *Zeitgeber* (literally, *time giver*) to maintain synchrony with the environment (entrainment), the capacity of the mechanism to persist under Midnight Sun conditions remains uncertain [4,5]. Climate change-induced latitudinal range shifts displace zooplankton such as *C. finmarchicus* to higher latitudes [6] yet the impact of high-latitude photoperiods on the endogenous timing systems of non-endemic species is currently unknown. Indeed, the northward expansion of organisms may be limited by the adaptive capacity of the clock to entrain to such extreme photoperiods [7,8].

The persistence of zooplankton DVM during the high Arctic Midnight Sun period is still debatable [9–13] and therefore raises the question whether associated clock gene oscillations are maintained at this time or whether the clock stops ‘ticking' and only reinitiates once clear light/dark cycles resume? Here we address this by determining circadian clock gene expression in *C. finmarchicus* during the Midnight Sun period.

## 2. Material and methods

### (a) Study area, field sampling and data collection

**Figure 1.**
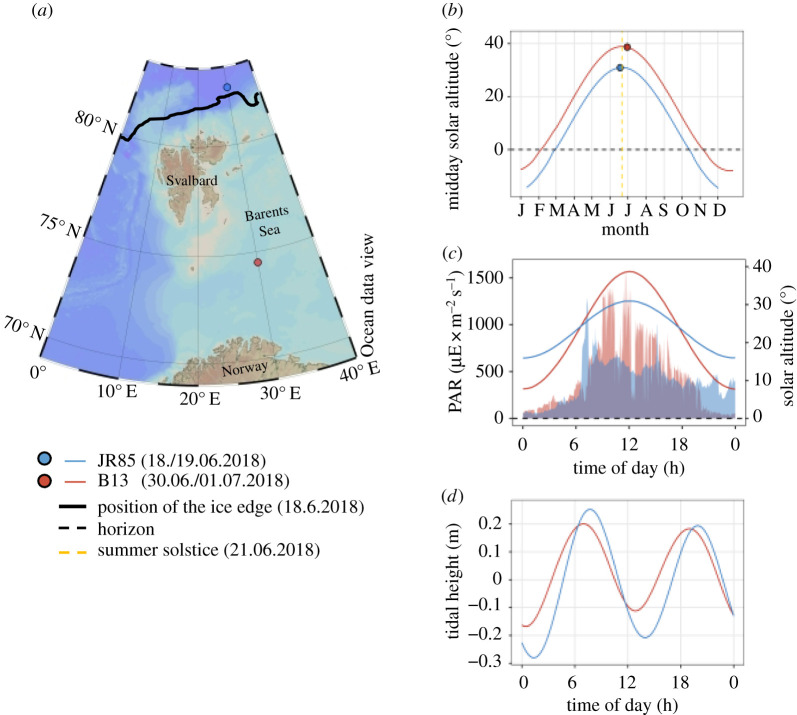
Physical characteristics of the sampling sites. (*a*) Map with sampled stations JR85 (blue) and B13 (red) and the position of the sea ice edge at the day of sampling at JR85 (18.06.2018). (*b*) Solar altitude at 12.00 throughout the year 2018 at both stations. The dashed yellow line marks the day of summer solstice, the dashed black line marks the horizon and the blue and red dots mark the day of sampling for the respective station. (*c*) Diel fluctuations in PAR (area plot) and solar altitude (lines) over the course of the first sampling day at each station (18.06.2018 and 30.06.2018 for JR85 and B13, respectively). (*d*) Tidal height over the course of the first sampling day at each station.

Sampling was conducted during Cruise JR17006 of the *RRS James Clark Ross* in summer 2018 at two stations along a latitudinal gradient, from the Nansen Basin ( JR85; 82.5° N, 30.85° E, sea ice-covered) to the southern Barents Sea (B13; 74.5° N, 30° E, sea ice-free, figure 1*a*). Sampling covered a complete 24 h cycle at 4 h intervals, resulting in seven timepoints per station. Sampling at JR85 started 3 days before the summer solstice, on 18th June at 11.00 and ended on 19th June at 11.00 (all times noted in local time (UTC + 2). Sampling at B13 started 9 days after the summer solstice, on 30th June at 14.00 and ended on 1st July at 14.00. For each timepoint, the water column was sampled between 200 m depth to the surface with a WP2 plankton net (200 µm mesh size). Net contents were preserved in RNA*later* (Ambion, UK) for later analysis post cruise.

Measurements of photosynthetically active radiation (PAR, i.e. the range of wavelengths available to photosynthesis, 400 to 700 nm) were taken by PQS1 PAR sensors (Kipp & Zonen, The Netherlands) from the ship's meteorological platform. Modelled data of sun altitude were obtained from the *United States Naval Observatory* (https://aa.usno.navy.mil/data/docs/AltAz.php, USNO, USA) and the *keisan.casio* website (https://keisan.casio.com/exec/system/1224682331). Information on the tidal dynamics have been drawn from the *TPX08* model [14] by using the *OTPS* package (Tidal Prediction Software, http://www-po.coas.oregonstate.edu/~poa/www-po/research/po/research/tide/index.html), via the *mbotps* program (MB-System; [15]). Additional methodological information and physical characteristics of the water column are available in the electronic supplementary material.

### (b) Copepod sorting and clock gene expression

For each replicate (*n* = 3–5 per time point), 15 *C. finmarchicus* CV stage copepods were sorted from the samples using morphological characteristics. Since there is considerable morphological overlap between congeners *C. finmarchicus* and *C. glacialis*, species identification was corroborated molecularly (see electronic supplementary material S1). Copepod total RNA was obtained by a combination of TRIzol-based extraction and the Direct-zol™ MiniPrep Kit (Zymo Research, USA). Total RNA was transcribed to cDNA using RevertAid H Minus Reverse Transcriptase (Thermo Scientific, USA). The expression of six core circadian clock genes (*clock*, *cycle*, *period1*, *timeless*, *cryptochrome2*, *vrille*), 2 circadian clock-related genes (*cryptochrome1* and *doubletime2*) and 3 candidate reference genes was determined using SYBRGreen-based quantitative real-time PCR (qPCR).

### (c) Data treatment and statistical analyses

Gene expression data were normalized according to the 2^−ΔCt^ method [16] using the geometric mean of *elongation factor 1α* and *16 s rRNA* as a reference. Profiles of clock genes were checked for rhythmic expression with ultradian (12 h ± 4 h) and daily (24 h ± 4 h) period ranges using the R package ‘RAIN’ [17]. Period phase estimates were obtained from the RAIN algorithm and the amplitude of oscillation was calculated by taking half the distance between the maximum and minimum expression value of each time series.

## 3. Results

During the sampling period, the sun remained permanently above the horizon (figure 1*b*) but still showed diel altitude cycles, reflected by changes in PAR (figure 1*c*). Daily PAR changes increased at the lower latitude and with time from the summer solstice. Both stations exhibited semi-diurnal tidal cycles. During the time of sampling at station JR85 (18–19/06/2018, 82.5° N, sea ice-covered), daily cycles in solar altitude were lower when compared to the time of sampling several days later at station B13 (30/06/2018 – 01/07/2018, 74.5° N, sea ice-free, figure 1*c*). Conversely, tidal height cycles were higher at JR85 when compared to B13 (figure 1*d*).

**Figure 2.**
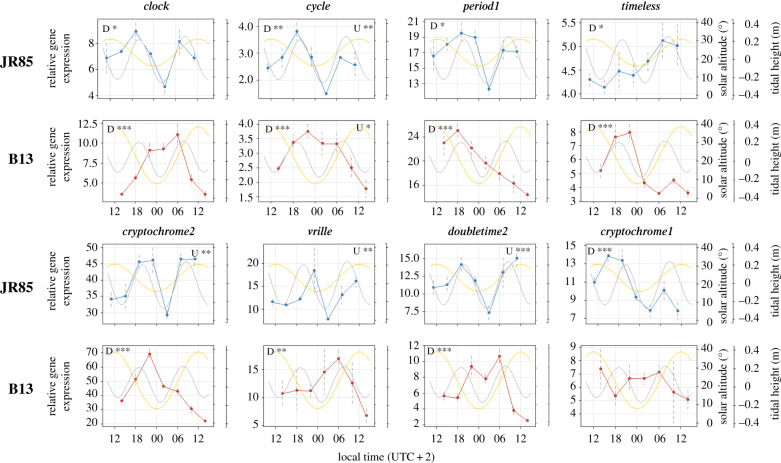
Temporal expression profiles of circadian clock and clock-related genes in CV stage *C. finmarchicus* during Midnight Sun in the high Arctic. Relative gene expression is shown in blue for the station JR85 (82.5̊ N, 18/19.06.2018) and in red for the station B13 (74.5̊ N, 30.06./01.07.2018). Grey dashed lines indicate the standard errors of the mean (s.e.m.). Significance levels of oscillations detected by RAIN (Benjamini–Hochberg-adjusted *p*-values) with daily (D, 24 ± 4 h) and ultradian (U, 12 ± 4 h) period ranges are indicated with stars: ‘*' adjusted-*p* ≤ 0.05, ‘**' adjusted-*p* < 0.01, ‘***' adjusted-*p* < 0.001. The yellow lines indicate the sun's altitude above the horizon and the grey lines the tidal height over the course of sampling.

The expression profiles of *C. finmarchicus* clock genes and clock-related genes showed significant oscillations at both stations (figure 2 and table 1). Rhythm analysis identified both daily (24 ± 4 h) and ultradian (12 ± 4 h) period ranges in gene expression, but with distinct differences between the stations. At station B13, all clock genes showed oscillations with daily periods, except for *cycle* (both daily and ultradian) and *cryptochrome1* (not significant). At station JR85, all clock genes showed significant oscillations but with an increase in ultradian periods. While *clock*, *period1*, *timeless* and *cryptochrome1* showed daily oscillations, *cryptochrome2*, *vrille* and *doubletime2* exhibited ultradian oscillations. As in B13, *cycle* showed both daily and ultradian oscillations in gene expression.

**Table 1.** Benjamini–Hochberg-adjusted *p*-values from rhythm analysis of clock gene expression profiles with RAIN for ultradian (12 ± 4 h) and daily (24 ± 4 h) period ranges. Values higher than *p* = 0.05 were considered not significant (n.s.).

target	JR85		B13	
	ultradian range	daily range	ultradian range	daily range
*clock*	n.s.	*0.01*	n.s.	<*0.0001*
*cycle*	*0.006*	*0.005*	*0.04*	<*0.0001*
*period1*	n.s.	*0.04*	n.s.	<*0.0001*
*timeless*	n.s.	*0.05*	n.s.	<*0.0001*
*cryptochrome2*	*0.004*	n.s.	n.s.	<*0.0001*
*vrille*	*0.002*	n.s.	n.s.	*0.001*
*doubletime2*	*0.0007*	n.s.	n.s.	<*0.0001*
*cryptochrome1*	n.s.	*0.0009*	n.s.	n.s.

## 4. Discussion

We reveal *in situ* daily circadian clock gene expression of a key zooplanktonic species, *C. finmarchicus,* at high Arctic latitudes (74.5° N, 82.5° N) during the Midnight Sun, near the time of the summer solstice. While limited studies have shown several Arctic species exhibit 24 h activity rhythms during the Polar Day [18–21], quite how the circadian clock is entrained without overt day/night cycles is unknown and currently under debate [4,5,22].

It is also still unclear what constitutes zooplankton DVM behaviour during this time, with some studies suggesting that synchronized DVM ceases [9–11] and some that it is maintained [12,13]. Copepods, specifically *C. finmarchicus*, are a dominant constituent of the zooplankton community and have been the focus of many DVM studies [2,12,23]. It has been shown that *C. finmarchicus* collected from a high-latitude Fjord (78° N) maintained circadian clock gene rhythmicity even under long photophases at the very end of the Midnight Sun period [24]. Our results go further, showing circadian clock gene oscillations within days of the summer solstice where daily changes in sun's altitude are at a minimum. At station B13 in the Southern Barents Sea (74.5° N, sea ice-free), clock gene expression shows pronounced daily oscillations and striking similarities with previous findings from animals at lower latitudes with *clock* and *period1* in antiphase [2]*.* While it is possible that self-sustained clock gene cycling could exist without synchronization to environmental cycles, the concordance of synchronicity between large numbers of individuals strongly suggests that the populations sampled are synchronized by a common *Zeitgeber*. Our results therefore strongly suggest that even small fluctuations in light intensity, barely perceptible to the human eye, are sufficient to sustain the circadian clock [22]. This is potentially explained by high irradiance [25] and spectral light sensitivity [26] in these organisms.

In contrast with the daily oscillations found at station B13, *C. finmarchicus* sampled at the northern sea ice-covered station JR85 (82.5° N) exhibited a significant increase of ultradian oscillations in circadian clock gene expression, with period ranges of 12 ± 4 h. The reduced daily solar altitude at JR85 is associated with less pronounced daily oscillations, lower amplitude and phase differences in some genes. For example, at JR85, *clock* peaks at decreasing light while at B13, it peaks at increasing light; however, *clock* and *period1* maintain their antiphase relationship. Furthermore, sampling at JR85 was conducted within very closely packed snow-covered sea ice, which will reduce the photoperiodic signal [27] thus limiting the potential of light to provide a reliable measure of time. It is noteworthy that the decrease in daily oscillations is not accompanied by a loss of rhythmicity but by the appearance of ultradian oscillations. These may be the result of circadian bimodality caused by complex interactions of multiple phase shifted circadian rhythms [28] or the presence of two circadian oscillators in different tissues peaking at different times of the day [29]. Further, ultradian rhythms of 12.4 h are often observed in marine organisms, including several crustaceans, under the influence of semi-diurnal tidal cycles [30,31]. Tides lead to cycles of current reversal, hydrostatic pressure, food, agitation or turbulence, known to entrain organisms [32–35]. In zooplankton, tidal rhythms of vertical migration [36–39] allow populations to maintain position within estuaries [36], while in *Pseudoclanaus* sp. cycles of ingestion have been documented under sea ice [39]. Here, the cyclic erosion of ice algae by tidal currents provided pulses of food for the copepods, with highest ingestion at slack water [39]. Our results reveal that ultradian oscillations of circadian clock genes at JR85 provide some correlation with tidal height cycles, though direct causation is untested (figure 2). Further, many covariables change with the tidal cycles, such as periodic turbulence, agitation or food supply. In the absence of overt photoperiodic cycles during the Midnight Sun period and under sea ice shading, tidal cues could function as an alternative *Zeitgeber* for the *C. finmarchicus* circadian clock and lead to both circadian and tidal oscillations of the circadian clock machinery [40,41]. Ultimately this would increase the adaptive advantages of a functioning clock in high-latitude environments, e.g. by optimizing the food intake and thus energy storage during the summer months. The accumulation of large lipid reserves throughout the spring/early summer is a fundamental process and key to *C. finmarchicus*' seasonal strategy to survive for the rest of the year in diapause and for a winter moult to adults [42]. An endogenous clock with sufficient plasticity to entrain to the extreme conditions at polar latitudes could therefore favour the permanent establishment of a boreal species like *C. finmarchicus* in the high Arctic.

## Supplementary Material

Supplementary Material 1: Methods

## Supplementary Material

Supplementary Material 2: Figure S1

## Supplementary Material

Supplementary Material 3. Table S1

## Supplementary Material

Supplementary Material 4. Table S2

## Supplementary Material

Supplementary Material 5. qPCR Data

## Data accessibility

Data supporting the paper are in the electronic supplementary material.
