# Supplementary material for: Evidence for oscillating circadian clock genes in the copepod Calanus finmarchicus during the summer solstice in the high Arctic
Source: Biol Lett. 2020 Jul 15;16(7):20200257. doi: 10.1098/rsbl.2020.0257 (PMC7423037; doi:10.1098/rsbl.2020.0257)
Supplement: Supplementary Material 1: Methods [file rsbl20200257supp1.docx]

**Evidence for oscillating circadian clock genes in the copepod *Calanus finmarchicus* during summer solstice in the high Arctic**

Lukas Hüppe, Laura Payton, Kim Last, David Wilcockson, Elizaveta Ershova, Bettina Meyer

Published in *Biology Letters*

**Supplementary Material 1: Methods**

**Study site characteristics**

During sampling time at JR85, the ice edge was located at about 81° to 82° N, roughly following the shelf slope north of Svalbard, thus station JR85 (82.56 °N) was located well within the ice cover, whereas station B13 (74.5 °N) was ice free. Information on the location of the sea ice edge at the time of sampling at JR85 were obtained from ice concentration maps available from the *meereisportal* [1].

The local physical properties of the water column were measured by vertical profiles of an SBE 911plus CTD (Sea-Bird Electronics, WA, USA). CTD profiles at station JR85 have been taken on 18^th^ June 15:45, and at B13 on 30^th^ June 12:30 (local time (UTC+2)). Temperature, pressure (depth), conductivity (salinity), oxygen saturation (SBE 43, Sea-Bird Electronics) and Chlorophyll *a* (Chl *a*) fluorescence (Aquatracka III fluorometer, Chelsea Technologies Group, UK) were measured. Profiles from the surface to 200 m depth (copepod sampling range) are shown in Supplementary Material 2, figure S1. Values remain largely constant below 200 m. CTD data were provided by the British Oceanographic Data Centre (BODC, UK).

**Sampling**

At each timepoint the water column has been sampled from 200 m to the surface with vertical hauls of a WP2 plankton net (opening ⌀: 57 cm, net length: 236 cm, mesh size: 200 µm) with a meshed bucket cod end (mesh size: 200 µm) at a speed of 0.5 m*s^-1^. Transferring the animals from the net into the stabilization solution was done within less than 12 minutes for all samplings. A ~12 h period of incubation at 2 - 4˚C has been allowed to soak the samples thoroughly with the RNA*later* stabilization solution (Ambion, UK) before they were transferred to -­80˚C for further transport and storage.

**Copepod sorting and species identification**

Copepods were sorted at 2˚C under a stereo microscope for species (*C. finmarchicus*) and stage (*CV*). To distinguish *C. finmarchicus* from its closely related congener *C. glacialis*, morphological indicators were used, in particular the redness of the antenna, which has been shown to be a good indicator in the regions of sampling [2]. However, since differences between the species can be very subtle, morphological identification was validated by molecular species identification on a subset of animals sorted from each station. Therefore, DNA was extracted from individual copepods using the HotShot method [3], and the species-specific nuclear insertion/deletion (InDel) marker G-150 was amplified using a modified protocol from Smolina et al. [4]. Identification was done by accessing the size of the resulting amplicon via electrophoresis on a 2% agarose gel. 99 % of the individuals identified as *C. finmarchicus* by the morphological identification method were also clearly identified as *C. finmarchicus* by the molecular identification method (n=305 individuals).

**RNA extraction and cDNA synthesis**

For each timepoint and replicate, 15 *C. finmarchicus CV* copepods were homogenized in 600 µl of TRIzol® reagent (ThermoFisher Scientific, USA) with a Precellys® 24 Tissue Homogenizer (Bertin Instruments, France). In total, 3-5 replicates per timepoint, and 5 replicates per timepoints, were analyzed at stations JR85 and B13 respectively.

For RNA extraction, a Phenol/Chloroform based single-step extraction in combination with a spin column based solid phase extraction (Direct-zol™ RNA MiniPrep Kit, Zymo Research, USA) has been used. To prevent gDNA contamination an on-column DNase I treatment has been applied as part of the RNA extraction kit. RNA purity and quantity have been checked on a NanoDrop 2000 spectrophotometer (ThermoFisher Scientific, USA) and RNA integrity was checked using a 2100 Bioanalyzer system (Agilent Technologies, USA).

2 µg of RNA was reverse transcribed using RevertAid H Minus Reverse Transcriptase (200 U/µl; ThermoFisher Scientific, USA) along with random pentadecamer primers (100 µM). cDNA synthesis has been performed under the following conditions: 8 mins. At 65°C (without RT), 5 mins. at 25˚C, 60 mins. at 42˚C, 5 mins. at 70˚C, ∞ at 4˚C.

**Gene expression analysis**

The expression of six circadian core clock genes (*clock*, *cycle*, *period1*, *timeless*, *cryptochrome2*, *vrille*), 2 circadian clock-related genes (*cryptochrome1*, involved in environmental synchronization; *doubletime2*, involved in period length modulation) and 3 candidate reference genes (*elongation factor 1-𝛼* , *RNA polymerase*, *16s rRNA*) was measured using a SYBRGreen based single gene assay. Primer sequences for clock genes and reference genes were designed using data from Christie et al. [5] and Lenz et al. [6] respectively, and are detailed in Supplementary Material 3, table S1.

Quantitative real-time PCR (qPCR) was conducted with the PowerUp™ SYBR® Green Master Mix (ThermoFisher, USA) on a ViiA^TM^ 7 system (Applied Biosystems, USA) using the following conditions: 1 cycle of 2 min at 50˚C and 2 min. at 95˚C (DNA polymerase activation), 40 cycles of 15 s at 95˚C and 1 min. at 60˚C (amplification of target cDNA), 1 cycle of 15 s at 95˚C (1.6˚C/sec), 1 min. at 60˚C (1.6˚C/sec) and 15 s at 95˚C (0.15˚C/sec; all steps for melting curve analysis).

**Normalization and Statistics**

The normalization of the gene expression levels was done with the 2^-∆Ct^ method [7], using the geometric mean of the most stable reference genes (*elongation factor 1-𝛼* and *16s rRNA)*. The 2^-∆Ct^ method is a mathematical method to quantify the expression level of a target gene relative to an internal standard, i.e. a reference gene, where Ct is the threshold cycle during qPCR and ΔCt = Ct_target gene_ - Ct_reference gene_. Temporal expression profiles of *C. finmarchicus* clock genes were checked for daily and ultradian rhythms by the non-parametric method “RAIN” [8] implemented in the R package “RAIN” and used within the environment of the statistical software R [9]. The time series have been tested using the “independent” mode. Significant rhythms with a period of 12 ± 4h were termed ultradian and significant rhythms with a period of 24 ± 4h were termed daily. For each period range and each gene, the wave form yielding the most significant result was chosen. The false discovery rate of the *p-values* was corrected using the Benjamini-Hochberg method [10], implemented in the *p.adjust* function in R. For genes displaying significant daily oscillations at both stations, phase estimates were obtained from the RAIN algorithm and the amplitude of oscillation was calculated by taking half the distance between the maximum and minimum expression value of each time series, as used by the ARSER algorithm [11].

For purposes of visualization, the fold change of the 2^-∆Ct^ values was calculated, by dividing each 2^-∆Ct^ value by the minimum value of the respective time series.

All graphs were produced using the ggplot2 package in R [12]. The map of the study site was created using Ocean Data View [13].

**Supplementary References**

1. Grosfeld K *et al.* 2016 Online Sea-Ice Knowledge and Data Platform. (doi:10.2312/polfor.2016.011)

2. Nielsen TG, Kjellerup S, Smolina I, Hoarau G, Lindeque P. 2014 Live discrimination of Calanus glacialis and C. finmarchicus females: can we trust phenological differences? *Mar. Biol.* **161**, 1299–1306. (doi:10.1007/s00227-014-2419-5)

3. Truett G e., Heeger P, Mynatt R l., Truett A a., Walker J a., Warman M l. 2000 Preparation of PCR-Quality Mouse Genomic DNA with Hot Sodium Hydroxide and Tris (HotSHOT). *BioTechniques* **29**, 52–54. (doi:10.2144/00291bm09)

4. Smolina I, Kollias S, Poortvliet M, Nielsen TG, Lindeque P, Castellani C, Møller EF, Blanco-Bercial L, Hoarau G. 2014 Genome- and transcriptome-assisted development of nuclear insertion/deletion markers for *Calanus* species (Copepoda: Calanoida) identification. *Mol. Ecol. Resour.* , n/a-n/a. (doi:10.1111/1755-0998.12241)

5. Christie AE, Fontanilla TM, Nesbit KT, Lenz PH. 2013 Prediction of the protein components of a putative *Calanus finmarchicus* (Crustacea, Copepoda) circadian signaling system using a de novo assembled transcriptome. *Comp. Biochem. Physiol. Part D Genomics Proteomics* **8**, 165–193. (doi:10.1016/j.cbd.2013.04.002)

6. Lenz PH, Roncalli V, Hassett RP, Wu L-S, Cieslak MC, Hartline DK, Christie AE. 2014 De Novo Assembly of a Transcriptome for Calanus finmarchicus (Crustacea, Copepoda) – The Dominant Zooplankter of the North Atlantic Ocean. *PLOS ONE* **9**, e88589. (doi:10.1371/journal.pone.0088589)

7. Livak KJ, Schmittgen TD. 2001 Analysis of relative gene expression data using real-time quantitative PCR and the 2(-Delta Delta C(T)) Method. *Methods San Diego Calif* **25**, 402–408. (doi:10.1006/meth.2001.1262)

8. Thaben PF, Westermark PO. 2014 Detecting Rhythms in Time Series with RAIN. *J. Biol. Rhythms* **29**, 391–400. (doi:10.1177/0748730414553029)

9. R Core Team. 2019 *R: A language and environment for statistical computing.* R Foundation for Statistical Computing, Vienna, Austria. See https://www.R-project.org/.

10. Benjamini Y, Hochberg Y. 1995 Controlling the False Discovery Rate: A Practical and Powerful Approach to Multiple Testing. *J. R. Stat. Soc. Ser. B Methodol.* **57**, 289–300.

11. Yang R, Su Z. 2010 Analyzing circadian expression data by harmonic regression based on autoregressive spectral estimation. *Bioinforma. Oxf. Engl.* **26**, i168-174. (doi:10.1093/bioinformatics/btq189)

12. Wickham H. 2009 *Ggplot2: Elegant Graphics for Data Analysis*. 2nd edn. Springer Publishing Company, Incorporated.

13. Schlitzer R. 2015 *Ocean data view. Available on https://odv.awi.de/*.
