# Supplementary material for: Evidence for oscillating circadian clock genes in the copepod Calanus finmarchicus during the summer solstice in the high Arctic
Source: Biol Lett. 2020 Jul 15;16(7):20200257. doi: 10.1098/rsbl.2020.0257 (PMC7423037; doi:10.1098/rsbl.2020.0257)
Supplement: Supplementary Material 2: Figure S1 [file rsbl20200257supp2.pptx]

## Slide 1
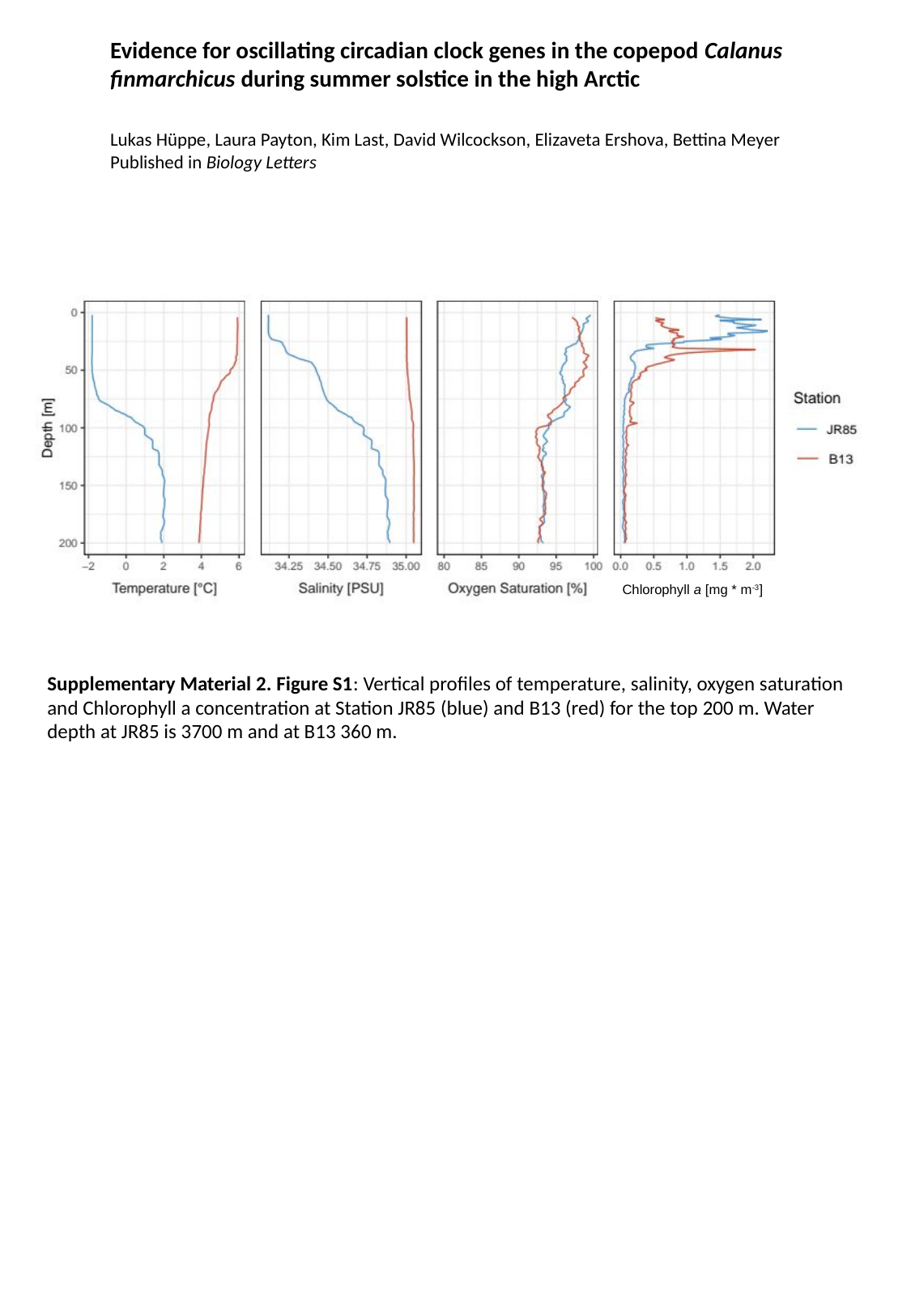

Evidence for oscillating circadian clock genes in the copepod Calanus finmarchicus during summer solstice in the high Arctic
Lukas Hüppe, Laura Payton, Kim Last, David Wilcockson, Elizaveta Ershova, Bettina Meyer
Published in Biology Letters
Chlorophyll a [mg * m-3]
Supplementary Material 2. Figure S1: Vertical profiles of temperature, salinity, oxygen saturation and Chlorophyll a concentration at Station JR85 (blue) and B13 (red) for the top 200 m. Water depth at JR85 is 3700 m and at B13 360 m.
