# Supplementary material for: Evidence for oscillating circadian clock genes in the copepod Calanus finmarchicus during the summer solstice in the high Arctic
Source: Biol Lett. 2020 Jul 15;16(7):20200257. doi: 10.1098/rsbl.2020.0257 (PMC7423037; doi:10.1098/rsbl.2020.0257)
Supplement: Supplementary Material 3. Table S1 [file rsbl20200257supp3.docx]

**Evidence for oscillating circadian clock genes in the copepod *Calanus finmarchicus* during summer solstice in the high Arctic**

Lukas Hüppe, Laura Payton, Kim Last, David Wilcockson, Elizaveta Ershova, Bettina Meyer

Published in *Biology Letters*

**Supplementary Material 3. Table S1**: Information on *C. finmarchicus* clock gene primer sequences used in this study, detailing function, direction, melting temperature (Tm), CG-content and the source transcriptome.

| Target | Target (abbr.) | Function | Direction | Sequence (5’ - 3’) | Tm (˚C) | CG (%) | Source |
| --- | --- | --- | --- | --- | --- | --- | --- |
| *clock* | *clk* | core clock | forward | ACTCGGATTGGCTTTGATGG | 65.6 | 50.0 | comp76772 c1 seq1  [5] |
|  |  |  | reverse | TTCTCAGGTGCAACGTTTCC | 64.7 | 50.0 |  |
| *cycle* | *cyc* |  | forward | CAGAGCAGGAAGGATAATGAGC | 63.5 | 50.0 | comp160482 c0 seq1  [5] |
|  |  |  | reverse | TGTAAGCATTGGCACTCAGC | 63.6 | 50.0 |  |
| *period 1* | *per1* |  | forward | ACATTGTCACAAGCCCTTGG | 64.4 | 50.0 | comp171214 c0 seq1  [5] |
|  |  |  | reverse | ACAGATGCTCCTTGTGATGC | 62.5 | 50.0 |  |
| *timeless* | *tim* |  | forward | CCTAACCTGTTACCGTTGACC | 61.8 | 52.4 | comp88114 c0 seq1  [5] |
|  |  |  | reverse | ATCGCTCACCAATGACTTCC | 63.6 | 50.0 |  |
| *cryptochrome 2* | *cry2* |  | forward | AGCAACCACCGAATATGACC | 63.2 | 50.0 | comp181328 c0 seq1  [5] |
|  |  |  | reverse | AACTGACCTTGTGGCATTCC | 63.5 | 50.0 |  |
| *vrille* | *vri* |  | forward | TGCAGCCTCACAACATTACC | 63.3 | 50.0 | comp71844 c0 seq1  [5] |
|  |  |  | reverse | AAACACGCAGGGATTTCACG | 66.6 | 50.0 |  |
| *doubletime 2* | *dbt2* | clock associated | forward | CAATGATACAGACTGGGACTGG | 62.9 | 50.0 | comp126103 c3 seq2  [5] |
|  |  |  | reverse | TGGTTGCATCTGACAGAACC | 63.4 | 50.0 |  |
| *cryptochrome 1* | *cry1* | light input pathway | forward | GGGTTTCAACTGGCTTTGG | 63.9 | 52.6 | comp37700 c0 seq1  [5] |
|  |  |  | reverse | CCTCTCACTTACCAGAAGATGC | 61.4 | 50.0 |  |
| *elongation factor 1-𝛼* | *ef1* | reference | forward | AGTTGCTGGCTTGTTCTTGG | 63.8 | 50.0 | comp8 c1 seq1  [6] |
|  |  |  | reverse | GGTTAAGTCCGTGGAGATGC | 63.0 | 55.0 |  |
| *RNA polymerase* | *rna-poly* |  | forward | TCAATGACGAGGTTCTCAGG | 62.5 | 50.0 | comp19535 c1 seq1  [6] |
|  |  |  | reverse | ATCAACTGTTGCCACTCTCG | 62.5 | 50.0 |  |
| *16s rRNA* | *16s* |  | forward | CCGCGTTAGTGTTAAGGTAGC | 62.1 | 52.4 | comp2 c0 seq1  [6] |
|  |  |  | reverse | CTTCTCGTCCTAGTACAACTGC | 59.3 | 50.0 |  |
