# Supplementary material for: Evidence for oscillating circadian clock genes in the copepod Calanus finmarchicus during the summer solstice in the high Arctic
Source: Biol Lett. 2020 Jul 15;16(7):20200257. doi: 10.1098/rsbl.2020.0257 (PMC7423037; doi:10.1098/rsbl.2020.0257)
Supplement: Supplementary Material 4. Table S2 [file rsbl20200257supp4.docx]

**Evidence for oscillating circadian clock genes in the copepod *Calanus finmarchicus* during summer solstice in the high Arctic**

Lukas Hüppe, Laura Payton, Kim Last, David Wilcockson, Elizaveta Ershova, Bettina Meyer

Published in *Biology Letters*

**Supplementary Material 4. Table S2**: Amplitude and phase of the expression profiles of genes displaying daily oscillations at both JR85 and B13. The amplitude was calculated for each selected gene at each station, by taking half the distance between the maximum and minimum value of expression. The phase estimates were obtained by RAIN and are given in local time (UTC+2).

|  | **JR85** | | **B13** | |
| --- | --- | --- | --- | --- |
| **Target** | Amplitude | Phase | Amplitude | Phase |
| *clock* | 2.1 | 19:00 | 3.7 | 06:00 |
| *cycle* | 1.2 | 19:00 | 1.0 | 22:00 |
| *period1* | 3.6 | 19:00 | 5.4 | 18:00 |
| *timeless* | 0.5 | 07:00 | 2.2 | 22:00 |
